# ADP-Induced Conformational Transition of Human Adenylate Kinase 1 Is Triggered by Suppressing Internal Motion of α_3_α_4_ and α_7_α_8_ Fragments on the ps-ns Timescale

**DOI:** 10.3390/biom12050671

**Published:** 2022-05-06

**Authors:** Chenyun Guo, Haoran Zhang, Weiliang Lin, Hanyu Chen, Ting Chang, Zhihua Wu, Jiaxin Yu, Donghai Lin

**Affiliations:** 1 Key Laboratory of Chemical Biology of Fujian Province, College of Chemistry and Chemical Engineering, Xiamen University, Xiamen 361005, China; guochy78@xmu.edu.cn (C.G.); 20520181152581@stu.xmu.edu.cn (W.L.); xmtonychen@163.com (H.C.); ctingmelody@163.com (T.C.); wuzh@xmu.edu.cn (Z.W.); yujiaxin2017@126.com (J.Y.); 2 Department of Pathogenic Biology, School of Basic Medicine, Tongji Medical College, Huazhong University of Science and Technology, Wuhan 430030, China; hrzhang@outlook.com

**Keywords:** *h*AK1, backbone dynamics, enzymatic activity, solution structure

## Abstract

Human adenylate kinase 1 (*h*AK1) plays a vital role in the energetic and metabolic regulation of cell life, and impaired functions of *h*AK1 are closely associated with many diseases. In the presence of Mg^2+^ ions, *h*AK1 in vivo can catalyze two ADP molecules into one ATP and one AMP molecule, activating the downstream AMP signaling. The ADP-binding also initiates AK1 transition from an open conformation to a closed conformation. However, how substrate binding triggers the conformational transition of *h*AK1 is still unclear, and the underlying molecular mechanisms remain elusive. Herein, we determined the solution structure of apo-*h*AK1 and its key residues for catalyzing ADP, and characterized backbone dynamics characteristics of apo-*h*AK1 and *h*AK1-Mg^2+^-ADP complex (holo-*h*AK1) using NMR relaxation experiments. We found that ADP was primarily bound to a cavity surrounded by the LID, NMP, and CORE domains of *h*AK1, and identified several critical residues for *h*AK1 catalyzing ADP including G16, G18, G20, G22, T39, G40, R44, V67, D93, G94, D140, and D141. Furthermore, we found that apo-*h*AK1 adopts an open conformation with significant ps-ns internal mobility, and Mg^2+^-ADP binding triggered conformational transition of *h*AK1 by suppressing the ps-ns internal motions of α_3_α_4_ in the NMP domain and α_7_α_8_ in the LID domain. Both α_3_α_4_ and α_7_α_8_ fragments became more rigid so as to fix the substrate, while the catalyzing center of *h*AK1 experiences promoted µs-ms conformational exchange, potentially facilitating catalysis reaction and conformational transition. Our results provide the structural basis of *h*AK1 catalyzing ADP into ATP and AMP, and disclose the driving force that triggers the conformational transition of *h*AK1, which will deepen understanding of the molecular mechanisms of *h*AK1 functions.

## 1. Introduction

Adenylate kinases (AKs) can read cellular adenine nucleotide balance and generate AMP molecules to trigger AMP signaling. Together with downstream AMP signaling (AK→AMP→AMP-sensors), AKs play fundamental roles in cell differentiation, cell polarity maintenance, and cell division [1,2,3,4]. As the crucial metabolic monitor of AMP signaling, the AKs family consists of nine major isoforms (AK1–AK9). AK1 is the major AMP generator and participates in many physiological processes [5,6]. The determined *E. coli* AK1 three-dimensional structure consists of LID, NMP, and CORE domains, and the LID and NMP domains are responsible for binding substrates, while the CORE domain governs the overall stability of the enzyme [7]. Furthermore, the CORE and NMP domains are conserved in different species whereas the LID domain is quite different. In the presence of Mg^2+^, AK1 can catalyze ATP and AMP into ADP. Due to ATP and AMP instabilities, analogs of ATP and AMP (AP_4_A, AP_5_A, AP_6_A) have been extensively used to exploit the interactions between AK1 and its substrates ATP and AMP [8]. The comparison of the crystal structures of *E.coli* AK1 in free form (PDB ID: 4AKE) and in complex with AP_5_A (PDB ID: 1AKE) displays that AK1 undergoes significant conformational change as both LID and NMP domains get close to the CORE domain when AK1 exerts its catalytic function [9,10]. Moreover, Pelz et al. demonstrate that AK1 binds ATP and AMP with an induced-fit mechanism in which substrate binding initiates AK1 transition from an open state to a closed state [11].

On the other hand, AK1 can reversibly catalyze ADP into ATP and AMP. Filippakopoulos et al. have determined the complex structure of *h*AK1 with the homologs of two ADP molecules, B_4_P (PDB ID: 2C95). The complex structure of *h*AK1-B_4_P indicates that *h*AK1 also adopts a closed conformation similar to the complex structure of *h*AK1-AP_5_A (PDB ID: 1Z83). The comparison of the apo-*Mtb*AK1 structure (PDB: 1P4S) with the *Mtb*AK1-Mg^2+^-ADP complex structure (PDB ID: 2CDN) illustrates that ADP binding also induces the conformational transition of AK1 from open to closed states [12,13]. Nevertheless, how substrate binding triggers the conformational transition of AK1 is still unclear. Herein, we determined the solution structure of *h*AK1 and identified key residues in *h*AK1 for catalyzing ADP by a combination of NMR chemical shift perturbation, enzyme activity assays, and point mutation experiments. Furthermore, we addressed the backbone dynamics characteristics of *h*AK1 when it exerted its catalytic function with NMR relaxation experiments. Our results are beneficial to exploring the AK1 catalytic cycle and providing a further mechanistic understanding of AK1 functions.

## 2. Materials and Methods

### 2.1. Preparation of Recombinant hAK1 and Its Mutants

Genes of *h*AK1 and its variants were separately cloned into the pGEX-6p-1 vector. Fusion proteins were expressed in *E. coli* BL21(DE3) at 22 °C and extracted by sonication, then purified by a GST affinity column. PreScission Protease was used to remove the GST tag at 4 °C. Finally, the target proteins were eluted into the NMR buffer (10 mM Na_2_HPO_4_, 100 mM Na_2_SO_4_, 50 mM NaCl, pH 6.2). Protein purities were analyzed by using 15 % (*w*/*v*) SDS-PAGE. Single-point mutations were conducted through PCR and verified by DNA sequencing from the Sangon Company.

### 2.2. Structure Calculation

Backbone and side-chain chemical shifts of apo-*h*AK1 have been assigned in our previous work (BMRB ID: 18133) [14]. ^1^H-^1^H NOE resonances in 3D ^13^C- and ^15^N-edited NOESY-HSQC spectra were manually and automatically assigned by using the Aria 2.3 software [15]. Resonance integrals of the NOESY-HSQC cross-peaks were used to generate ^1^H-^1^H distance restraints. Backbone dihedral restraints were predicted with the TALOS-N program based on assigned chemical shifts of *h*AK1 [16]. Annealing parameters were optimized for the final run (20,000, 1000, 120,000, and 96,000 for high temperature, refinement, cool1, and cool2 steps). Ultimately, 300 models of *h*AK1 were calculated and refined with Aria 2.3, from which 20 lowest-energy structures were chosen as the final conformational ensemble. The PROCHECK program was used to assess the structural quality of the *h*AK1 protein [17].

### 2.3. NMR Chemical Shift Perturbation Experiments

NMR titration experiments were conducted on Bruker Avance Ⅲ 600 MHz spectrometer (Bruker, Billerica, MA, USA) at 298K. The apo-*h*AK1 sample was solved in NMR buffer added with 5 mM EDTA. The holo-*h*AK1 sample was solved in NMR buffer added with 5 mM MgCl_2_, and ADP was titrated step-by-step according to the following molar ratios of protein: ligand: 1:0, 1:0.5, 1:1, 1:2, 1:3, 1:4, 1:5, 1:6, 1:7, 1:8. We calculated chemical shift perturbations (∆δ) of *h*AK1 at the molar ratio of 1:8, using the following formula:

(1) Δδ=Δδ2(H 1)+Δδ2(N 15)/25,

where ∆δ(^1^H) is the perturbation in proton and ∆δ(^15^N) is the perturbation in nitrogen dimension.

### 2.4. Enzymatic Activity Assay

Enzymatic activities of *h*AK1 and its mutants were assessed in a coupling reaction with hexokinase-glucose-6-phosphate dehydrogenase, and the fluorescence intensity of final product NADPH at 456 nm was proportional to the enzymatic activity of *h*AK1 [18]. In detail, the enzymatic activities were measured in a reaction solution (50 mM Tris-HCl, 7.2 mM MgCl_2_, 2 mM Glucose, 1.64 mM NADP, 500 µg/L BSA, 3 mM ADP, 0.1 ug/mL *h*AK1, and 0.7 U/mL hexokinase-glucose-6-phosphate dehydrogenase) at 37 °C for 5 min. Then, a stop solution (10 mM NaOH, 5 mM EDTA) was added into the reaction solution to stop the reaction.

### 2.5. Backbone Relaxation Measurements

Relaxation experiments were conducted on Bruker Avance Ⅲ 600 MHz spectrometer at 298 K. Standard pulse sequences were used to obtain longitudinal and transverse relaxation rates *R*_1_ and *R*_2_, as well as {^1^H}-^15^N heteronuclear steady-state NOEs (hnNOEs). For ^15^N T_1_ measurements, the delay times were set to 0.01, 0.05, 0.1, 0.2, 0.4, 0.8, 1.2, 1.5, and 1.8 s in random order. For ^15^N T_2_ measurements, the delay times were set to 16.96, 33.92, 50.88, 67.84, 84.80, 101.76, 118.72, 135.68, 152.64, and 186.56 ms in random order. Two delay times were duplicated for both T_1_ and T_2_ experiments at the end of the experiments to estimate uncertainties of T_1_ and T_2_ values. For hnNOE experiments, a delay of 2 s was followed by ^1^H saturation of 3 s, and in the control experiments without ^1^H saturation, a total delay of 5 s was applied. The relaxation data were processed and analyzed with Bruker Dynamics Center 2.3 (Bruker, Billerica, MA, USA).

### 2.6. Model-Free Analysis

Model-free analysis of relaxation parameters *R*_1_, *R*_2_, and hnNOE was performed with the TENSOR2 and FAST-Modelfree programs following the Lipari-Szabo formalism [19,20,21,22]. Internal motions of backbone amide groups were fitted to five different model-free models described with a combination of *S*^2^, *τ_e_*, and *R_ex_*. *S*^2^ is the squared order parameter, *τ_e_* is the correlation time of the internal motion (ps-ns time scale), and *R_ex_* is the chemical exchange rate (μs-ms time scale) contributing to *R*_2_. An axially symmetric diffusion model was chosen for the model-free calculations. The *F*-test statistics were used to select the model satisfying the data with the lower number of parameters.

### 2.7. Reduced Spectral Density Mapping

Reduced spectral density mapping was conducted on the obtained relaxation data *R*_1_, *R*_2_, and hnNOE [23]. These data were converted into three *J*(ω) values *J*(0), *J*(ω_N_), *J*(0.87ω_H_) using the script reported by Leo Spyracopoulos [24].

## 3. Results

### 3.1. NMR Solution Structure of hAK1

**Figure 1:**
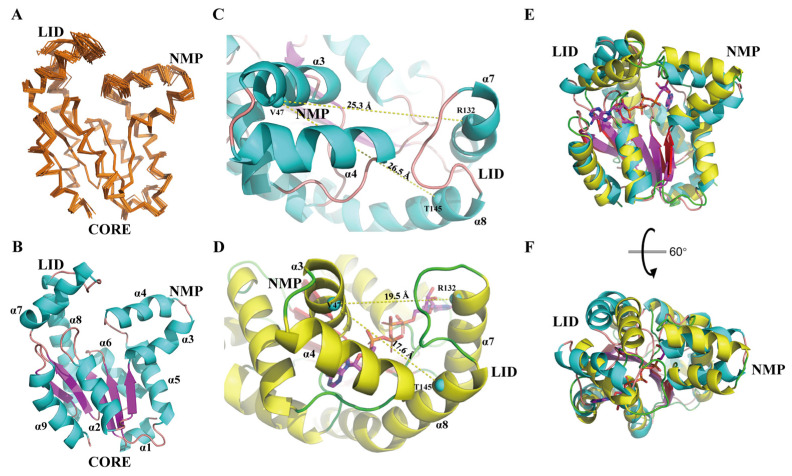
Standalone and comparative representation of the apo-*h*AK1 structure. (**A**) Superposition of 20 lowest-energy structures of apo-*h*AK1 represented as ribbons (PDB ID: 7X7S). (**B**) Mean structure of apo-*h*AK1 depicted in cartoon. α-helices and β-strands are colored in cyan and purple, respectively. (**C**) Conformation of NMP and LID domains in apo-*h*AK1. (**D**) Conformation of NMP and LID domains in the *h*AK1-B_4_P complex (PDB ID: 2C95). The C_α_ atoms of V47 in α_3,_ R132 in α_7_ and T145 in α_8_ are selected to optimally exhibit the distances between α_3_ and α_7_ or α_8_, which are displayed as cyan spheres. The α-helices of apo-*h*AK1 and B_4_P-complexed *h*AK1 are colored in cyan and yellow, respectively. (**E**,**F**) Superposition of 7X7S (α-helices in cyan and β-strands in purple) and 2C95 (α-helices in yellow and β-strands in red) structures. 7X7S atom coordinates were RMS fitted to 2C95 by C_α_ atoms in α_1_, α_2_, α_5_, α_6_, α_9_, and β_1_-β_5_ for visualization.

To disclose molecular mechanisms underlying *h*AK1 catalyzing ADP, we determined the solution structure of *h*AK1 using heteronuclear multi-dimensional NMR spectroscopy. The final structure ensemble consists of 20 lowest-energy models (Figure 1A), and the NMR restraints and structure statistics are shown in Table 1. Structural quality analysis by the PROCHECK program illustrated that most of the modeled residues were in preferred and allowed regions (Table 1). The root-mean-square deviation (RMSD) for the 20 structural models to the mean structure reached 0.57 Å and 0.84 Å for backbone atoms and heavy atoms, respectively, indicating a well-defined structure ensemble. The solution structure of *h*AK1 has been deposited in the Protein Data Bank with an accession code of 7X7S.

Overall, *h*AK1 adopted a globular fold comprising 9 α-helices (α_1_: 2–7, α_2_: 21–32, α_3_: 39–49, α_4_: 52–63, α_5_: 69–83, α_6_: 99–109, α_7_: 122–135, α_8_: 143–167, α_9_: 179–193) and a five-stranded β-sheet (β_1_: 9–14, β_2_: 35–38, β_3_: 89–83, β_4_: 114–119, β_5_: 170–174) (Figure 1B). Three domains, namely NMP, LID, and CORE were identified in the *h*AK1 protein: NMP contained α_3_ and α_4_; LID consisted of α_7_ and α_8_; CORE was represented by the β-sheet surrounded by other helices. Notably, NMP and LID domains exhibited larger RMSDs than the global average due to relatively fewer NOE restraints (Figure 1A), indicative of their structural flexibilities in solution.

To investigate structural alterations of *h*AK1 upon binding to its substrates, we compared the three-dimensional structures of apo-*h*AK1 (PDB ID: 7X7S) and *h*AK1-B_4_P complex (PDB ID: 2C95). Expectedly, NMP and LID domains exhibited noticeable conformational changes. The NMP and LID domains adopted an open conformation in apo-form, characterized by a relatively large distance between α_3_ and α_7_ or α_8_ of 25.3 Å or 26.5 Å, respectively (Figure 1C). Upon B_4_P binding to *h*AK1, both NMP and LID domains moved inward, resulting in reduced corresponding distances of 19.5 Å or 17.6 Å, respectively (Figure 1D). Furthermore, the local structural alignment of apo-*h*AK1 with B_4_P-complexed *h*AK1 gave an RMSD of 1.73 Å for the CORE domain, 5.17 Å for the region consisting of NMP and LID domains (Figure 1E–F). Consequently, B_4_P binding to *h*AK1 led to a “closed” conformation for the NMP and LID domains.

### 3.2. Key Residues for hAK1 Catalyzing ADP

**Figure 2:**
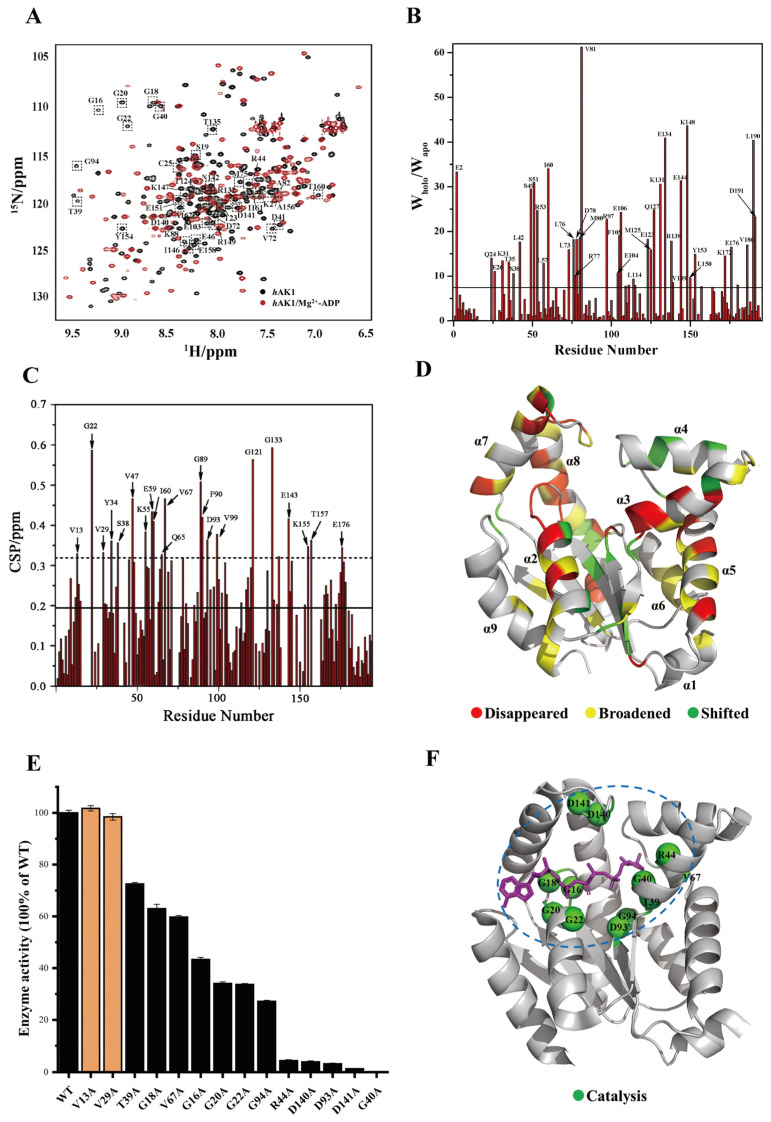
Determination of key residues for *h*AK1 catalyzing ADP. (**A**) Overlaid 2D ^1^H-^15^N HSQC spectra of the *h*AK1 protein with and without Mg^2+^-ADP addition. Black: *h*AK1, Red: *h*AK1-Mg^2+^-ADP; dashed box represents disappeared peaks after Mg^2+^-ADP addition. (**B**) Identification of residues with significant peak broadening after Mg^2+^-ADP addition. (**C**) Identification of residues with significant peak shifting after Mg^2+^-ADP addition. (**D**) Mapping perturbed residues onto the three-dimensional structure of apo-*h*AK1 (PDB ID: 7X7S). (**E**) Enzymatic activity comparison of *h*AK1 and its variants. (**F**) Mapping key residues for *h*AK1 catalyzing ADP onto the 3D structure of the *h*AK1-B_4_P complex (PDB ID: 2C95). Blue dashed circle: ADP catalyzing center.

As reported previously, *h*AK1 can efficiently catalyze mutual transformation among ADP, ATP, and AMP only in the presence of some divalent ions such as Mg^2+^ [25]. To identify key residues for *h*AK1 catalyzing ADP, we performed NMR titration experiments on ^15^N-labeled *h*AK1 with increasing ADP concentration in the presence of Mg^2+^. It was previously reported that *h*AK1 is associated with five statuses during its catalytic cycle: open, partially open, intermediate, partially closed, and closed [26]. Expectedly, ADP titration resulted in greater conformational exchange for *h*AK1. About 50% of peaks in the 2D ^1^H-^15^N HSQC spectra of *h*AK1/Mg^2+^-ADP were appreciably perturbed, including 36 vanished peaks, 40 broadened peaks and 21 shifted peaks (Figure 2A–C). These perturbed residues were identified and mapped into the three domains of apo-*h*AK1 based on the backbone assignments we previously completed [14], suggesting that Mg^2+^-ADP caused a significant change to the whole structure of apo-*h*AK1 (Figure 2D). In order to identify key residues for *h*AK1 catalyzing ADP, we mutated 14 significantly perturbed residues, which were situated on the *h*AK1-B_4_P interface identified from the 3D structure of *h*AK1 complexed with B_4_P (PDB ID: 2C95), including G16, G18, G20, G22, T39, G40, R44, Q65, V67, D93, G94, R132, D140, and D141. Besides, we also mutated significantly perturbed residues V13 and V29, which were located far away from the binding interface.

The enzymatic activities of 12 *h*AK1 mutants have decreased. As shown in Figure 2E, five mutations (G40A, R44A, D93A, D140A, and D141A) remarkably decreased enzymatic activities of *h*AK1 by more than 90%. Moreover, four mutations (G16A, G20A, G22A, and G94A) significantly decreased enzymatic activities of *h*AK1 by more than 50%. Furthermore, three mutations (G18A, T39A, and V67A) reduced enzymatic activities of *h*AK1 by 20%–40%. Thus, we identified 12 key residues for *h*AK1 catalyzing ADP, including G16, G18, G20, G22, T39, G40, R44, V67, D93, G94, D140, and D141, which are highlighted in the 3D structure of the *h*AK1-B_4_P complex (Figure 2F). Notably, V13A and V29A mutations did not significantly decrease the enzymatic activity of *h*AK1, implying that their chemical shift perturbation was primarily caused by the overall conformational change of *h*AK1 upon addition of ADP.

### 3.3. Comparison of Relaxation Data between apo-hAK1 and holo-hAK1

As it is known, dramatic conformational changes occurred during the *h*AK1 catalytic cycle. Although structural studies have provided ample evidence for conformational changes of *h*AK1, few systematic experimental studies were reported to explore dynamic properties of the *h*AK1 enzyme through its catalytic cycles [27,28]. To disclose the driving force in the conformational transition of *h*AK1, we determined backbone dynamics parameters of apo-*h*AK1 and holo-*h*AK1 (*h*AK1-Mg^2+^-ADP), including ^15^N longitudinal relaxation rates (*R*_1_), ^15^N transverse relaxation rates (*R*_2_) and {^1^H}-^15^N heteronuclear steady-state NOEs (hnNOE).

**Figure 3:**
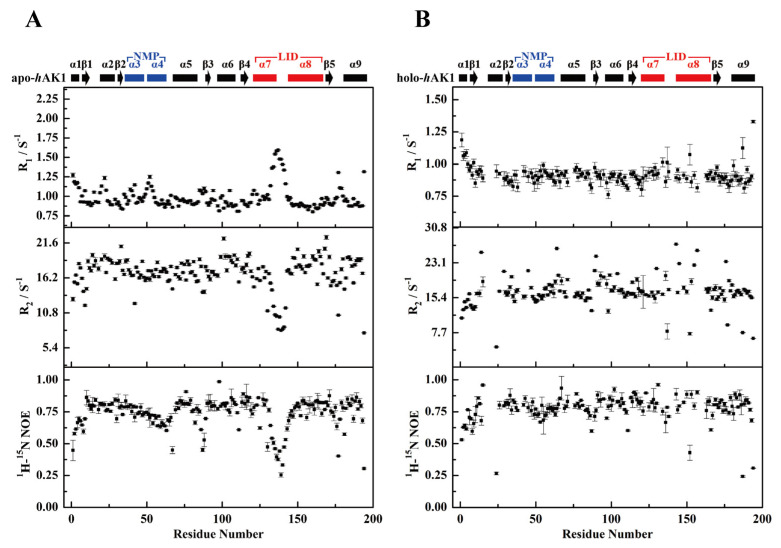
*R*_1_, *R*_2_, and hnNOE values of apo-*h*AK1 (**A**) and holo-*h*AK1 (**B**). The secondary structure elements of *h*AK1 are displayed on the top of the figure. The α_3_α_4_ in the NMP domain and α_7_α_8_ in the LID domain are highlighted in blue and red, respectively.

The mean *R*_1_ and *R*_2_ values of apo-*h*AK1 were 0.983 ± 0.007 s^−1^ and 16.941 ± 0.178 s^−1^, respectively (Figure 3A). After Mg^2+^-ADP addition, the mean *R*_1_ and *R*_2_ values became 0.917 ± 0.041 s^−1^ and 16.709 ± 0.329 s^−1^, respectively. Overall, Mg^2+^-ADP binding did not result in significant changes in mean relaxation rates for *h*AK1 (Figure 3A–B). However, holo-*h*AK1 displayed greater local fluctuations of the backbone relaxation parameters compared to apo-*h*AK1, especially in the α_3_α_4_ fragment (residues 39–72) of the NMP domain and the α_7_α_8_ fragment (residues 120–152) of the LID domain. Both α_3_α_4_ and α_7_α_8_ fragments of apo-*h*AK1 exhibited higher *R*_1_ values and lower *R*_2_ values than the average values, implying that these regions might undergo significant internal motions on the ps-ns time scale with observable structural flexibility. Differently, holo-*h*AK1 showed relatively stable *R*_1_ and *R*_2_ values across the amino acid sequence with insignificant ps-ns internal motions in α_3_α_4_ and α_7_α_8_ fragments. Besides, α_7_α_8_ of holo-*h*AK1 displayed profoundly increased {^1^H}-^15^N NOE values, suggesting that the LID domain became more rigid upon Mg^2+^-ADP binding. Therefore, we speculate that Mg^2+^-ADP binding remarkably changed the dynamics properties of *h*AK1 by suppressing the ps-ns internal motions of α_3_α_4_ and α_7_α_8_.

### 3.4. Model-Free Analysis

The relaxation data of *R*_1_, *R*_2_, and {^1^H}-^15^N NOE were further processed by using model-free analysis. We firstly obtained rotational diffusion tensors describing the overall tumbling of apo-*h*AK1 and holo-*h*AK1. The best-fit χ^2^ value of the axially symmetric diffusion model was 291.2 for apo-*h*AK1 and 317.3 for holo-*h*AK1, indicative of well-fitted models. The estimated rotational correlation time (*τ_m_*) was 13.929 × 10^−9^ s for apo-*h*AK1 and 13.406 × 10^−9^ s for holo*-h*AK1. The diffusion tensor ratio *D*_//_/*D*_⊥_ was 1.190 for apo-*h*AK1 and 1.149 for holo*-h*AK1, indicating that internal motions of both apo-*h*AK1 and holo*-h*AK1 could be described by using slightly prolate diffusion models.

**Figure 4:**
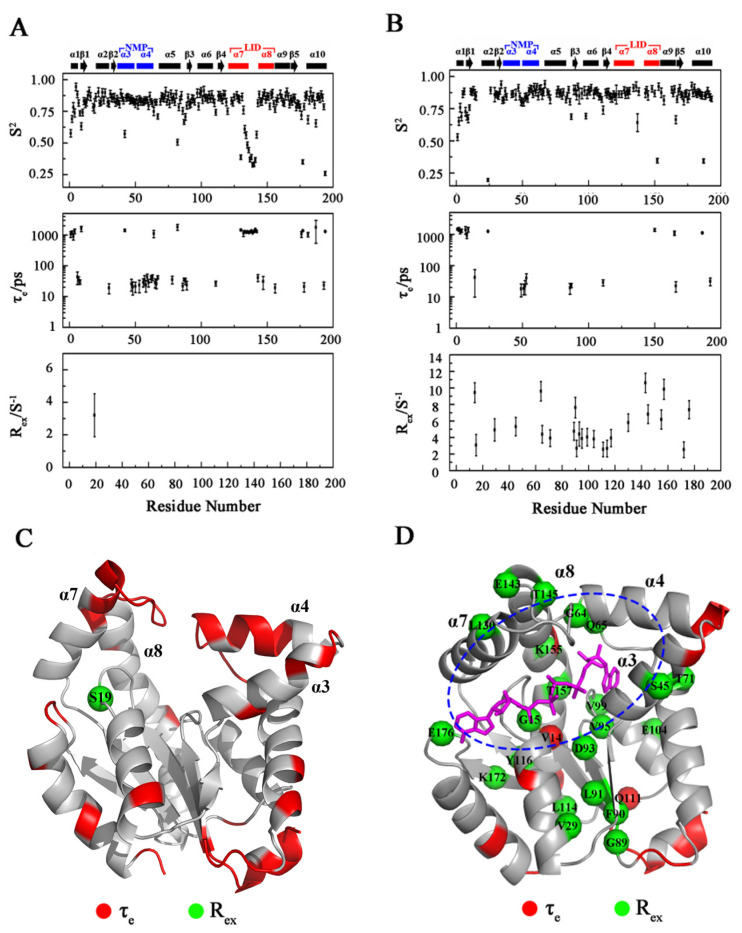
Model-free analyses of apo-*h*AK1 and holo*-h*AK1. (**A**,**B**) Three dynamics parameters (*S*^2^, *τ_e_* and *R_ex_*) fitted from model-free analyses based on the obtained relaxation parameters of *R*_1_, *R*_2_, and {^1^H}-^15^N NOE of apo-*h*AK1 (**A**) and holo-*h*AK1 (**B**). (**C**,**D**) Residues with *τ_e_* (displayed in red) and *R_ex_* (showed as green balls) are mapped onto the 3D structures of apo-*h*AK1 (**C**) and *h*AK1-Mg^2+^-B_4_P (**D**). Red balls: residues with *τ_e_* and *R_ex_* simultaneously. Blue dashed circle: the catalyzing center. The secondary structure elements of *h*AK1 are displayed on the top of the figure. The α_3_α_4_ in the NMP domain and α_7_α_8_ in the LID domain are highlighted in blue and red, respectively.

The model-free analysis of relaxation data provided an internal correlation time (*τ_e_*), and an order parameter (*S*^2^) as well as a conformational exchange rate (*R_ex_*) for describing backbone dynamics characteristic of each residue for the protein. Results derived from the model-free analysis of apo-*h*AK1 showed that residues with smaller *S*^2^ and larger *τ_e_* values were primarily distributed in the α_3_α_4_ and α_7_α_8_ fragments, indicating that these two fragments had apparent fast internal mobility on the ps-ns timescale (Figure 4A). However, once Mg^2+^-ADP binding, these prominent ps-ns internal motions were attenuated, even quenched in the α_3_α_4_ and α_7_α_8_ as shown by increased *S*^2^ and decreased *τ_e_* values (Figure 4B). On the other hand, backbone amide groups of apo-*h*AK1 did not show significant slow internal motions on the µs-ms timescale, as indicated by only one residue exhibiting an observable *R_ex_* value (Figure 4C). The Mg^2+^-ADP binding brought significant µs-ms internal motion in holo-*h*AK1, as reflected by 24 residues with large *R_ex_* values, which surrounded the catalyzing center including V14, G15, V29, S45, G64, Q65, T71, G89, F90, L91, D93, Y95, V99, E104, Q111, L114, Y117, L130, E143, T145, K155, T157, K172, and E176 (Figure 4D). Notably, the LID domain exhibited promoted conformational exchange and structural flexibility, which was potentially favorable for *h*AK1 exerting its catalytic functions. 

**Figure 5:**
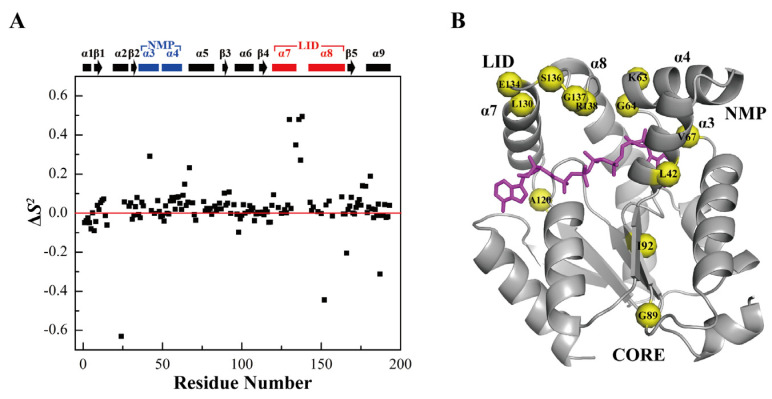
Comparison of the *S*^2^ values of holo-*h*AK1 with apo-*h*AK1. (**A**) Differences of the *S*^2^ values between holo-*h*AK1 and apo-*h*AK1. The secondary structure elements of *h*AK1 are displayed on the top of the figure. The α_3_α_4_ in the NMP domain and α_7_α_8_ in the LID domain are highlighted in blue and red, respectively. (**B**) Residues in holo-*h*AK1 with increased *S*^2^ values are mapped onto the 3D structure of the *h*AK1-Mg^2+^-B_4_P complex.

As it is known, AK1 binds substrates in the open conformation, catalyzes the phosphor-transfer reaction in the closed conformation, and releases products again in the open conformation. The holo*-h*AK1 adopts a closed conformation upon Mg^2+^-ADP binding, which is helpful for avoiding hydrolysis of the product ATP. Note that some residues showed relatively larger *S*^2^ values, including E26, Y34, G89, and I92 located in the CORE domain, and A120, L130, E134, S136, G137, and R138 in α_7_α_8_ of the NMP domain, as well as K63, G64, and V67 in α_3_α_4_ of the LID domain. These regions displayed relatively rigid dynamics properties, which were potentially responsible for restricting the conformational exchange required for *h*AK1 binding more efficiently Mg^2+^-ADP (Figure 5).

### 3.5. Reduced Spectral Density Mapping

**Figure 6:**
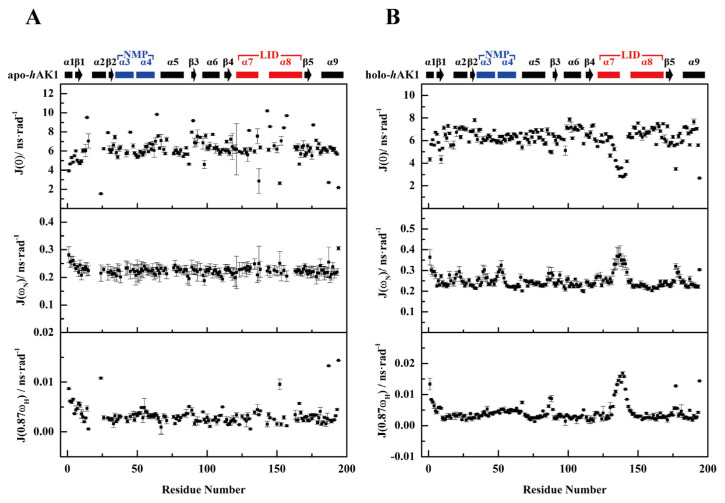
Spectral density functions of residues in apo-*h*AK1 (**A**) and holo-*h*AK1 (**B**). Analysis of reduced spectral density mapping was carried out using Mathematica notebooks from Leo Spyracopoulos [24]. The secondary structure elements of *h*AK1 are displayed on the top of the figure. The α_3_α_4_ in the NMP domain and α_7_α_8_ in the LID domain are highlighted in blue and red, respectively.

Reduced spectral density functions at three frequencies *J*(0), *J*(ω_N_) and *J*(0.87ω_H_) were calculated for each residue based on the obtained ^15^N relaxation parameters of apo-*h*AK1 and holo-*h*AK1. As expected, both α_3_α_4_ and α_7_α_8_ fragments displayed different spectral density functions, similar to the above-described relaxation parameters (Figure 6).

**Figure 7:**
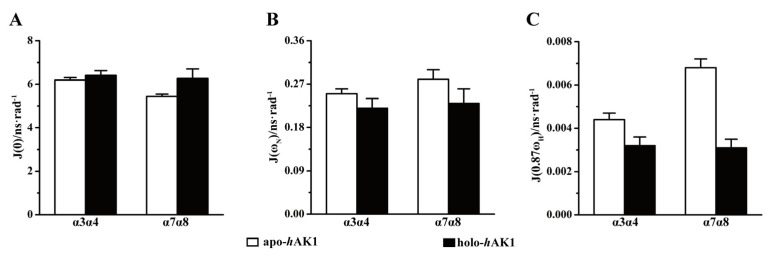
*C*omparison of spectral density functions of the α_3_α_4_ and α_7_α_8_ fragments between apo-*h*AK1 and holo*-h*AK1. (**A**) *J*(0); (**B**) *J*(ω_N_); (**C**) *J*(0.87ω_H_).

Significantly, Mg^2+^-ADP binding to *h*AK1 increased average *J*(0) values in the α_3_α_4_ and α_7_α_8_ fragments (Figure 7A), suggesting that both fragments had enhanced internal mobility on the µs-ms timescale. Furthermore, Mg^2+^-ADP binding also remarkably decreased average *J*(ω_N_) and *J*(0.87ω_H_) values for α_3_α_4_ and α_7_α_8_, indicative of suppressed structural flexibility on the ps-ns timescale (Figure 7B,C). It seemed that despite the overall 3D structure of holo-*h*AK1 being more rigid than apo-*h*AK1, Mg^2+^-ADP binding profoundly promoted the µs-ms conformational exchange which potentially facilitated catalysis reaction and conformational transition. These results were basically consistent with those from the model-free analysis (Figure 4).

## 4. Discussion

We have determined the solution structure of apo-*h*AK1, and identified 12 key residues for *h*AK1 catalyzing ADP, including G16, G18, G20, G22, T39, G40, R44, V67, D93, G94, D140, and D141. Both model-free analysis and spectral density analysis showed that apo-*h*AK1 adopts an open conformation with significant ps-ns internal mobility, thereby allowing local structural rearrangements to accommodate *h*AK1 binding Mg^2+^ and ADP. The Mg^2+^-ADP binding substantially triggers the conformational transition of *h*AK1 by suppressing the fast internal motions of α_3_α_4_ in the NMP domain and α_7_α_8_ in the LID domain, and simultaneously promoting the slow internal motions of the protein. Upon *h*AK1 binding Mg^2+^ and ADP, both α_3_α_4_ and α_7_α_8_ become more rigid so as to fix the substrate, while the catalyzing center of *h*AK1 experiences promoted µs-ms conformational exchange, potentially facilitating catalysis reaction and conformational transition.

While our study suggests several potential determinants of *h*AK1’s catalyzing functionality from a view of structural biology and biophysics, certain considerations arising from our interpretation of the results need in-depth discussion. For example, because of the inherent conformation averaging effect of the in-solution NMR sampling, the rare conformations existing at the saddle points on the free energy landscape might be omitted during structure calculation and, thus, are not necessarily present in the final NMR ensemble. Expectedly, MD simulations would be helpful to remedy the lack of atom-level portrayal of important conformations, which are calculated based on NMR-derived constraints. MD simulations can catch vital biomolecular processes by revealing atomic coordinates at fs-level resolution, and predict how protein dynamics respond to both perturbations imposed on residues and introduction of ligands [29]. Recent advances in accelerated MD might particularly enhance the sampling of biomolecular binding events on an enlarged simulation timescale than the conventional ns-μs all-atom simulation [30]. The advanced MD simulation methodology will allow for the thorough characterization of lid motion and substrate binding thermodynamics and kinetics of *h*AK1.

Another critical question lies before this study about how the internal motion of α_3_α_4_ and α_7_α_8_ fragments on the ps-ns timescale induces the biochemically significant steps that occur on the µs-ms timescale. Previous studies reported that the adenylate kinase from *Aquifex aeolicus* (*Aquifex* Adk) exhibits a free-energy landscape with multiple kinetic states along the reaction pathway during the catalysis process [31], and the ligand-free *Aquifex* Adk adopts different conformations with variable degrees of the closure of lids [32]. Some of the flexible “hotspot” residues with backbone conformational alteration allowing for motif-wise lid motion, have been identified as the “hinges”, thereby raising an assumption that the amplitude of the fast internal motion of the hinges on the ps-ns timescale is mechanistically responsible for the biocatalytically significant lid motions on the μs-ms timescale [33]. Furthermore, molecular dynamics (MD) simulation of a sextuple mutant of *Aquifex* Adk supports this assumption by characterizing the motion trajectories at an atomic resolution [33]. In this mutant, the non-conservative backbone-rigidifying prolines and π-π-stacking-inducing phenylalanines were replaced with the corresponding residues of an Adk homolog with lower NMR-derived *S*^2^ values for hinge residues. In other words, the closure of lids might result from “individual attempts by local groups to overcome the energy barrier for the conformational transition” [33]. Due to the domain-wise structural and functional similarities between *Aquifex* Adk and *h*AK1, we, therefore, conjecture that the above analyses for *Aquifex* Adk is applicable to a high extent for *h*AK1.

Herein, we found that 12 key residues located at the binding interface of *h*AK1 with ADP are important to *h*AK1 catalytic activity based on their chemical shift perturbation after ADP addition and their enzymatic change after mutation. However, the nature of the mutation-induced catalytic activity loss in detail remains to be explored. A mechanism mediated by the mis-oriented Mg-ADP interaction is conceivable, but, in the meantime, binding incapability as a result of the misfold of *h*AK1 is also a possibility. Expectedly, a combination of our biophysical analysis with the high-precision MD simulations might better illustrate the motional patterns of the mutants, thus facilitating the molecular mechanism underlying the catalytically relevant residues. What is more, we also found that five residues (L43, M61, L66, R128 and V182), which were located at the binding interface of *h*AK1, did not show significant chemical shift perturbations in the NMR titration experiments, suggesting that they were not greatly involved in Mg-ADP binding to *h*AK1. We, thus, speculate these residues were not closely related to the catalytic function of *h*AK1. However, mutation experiments and catalytic activity measurements are needed to perform in the future to examine whether these residues are catalytic important for *h*AK1.

This study mainly focused on biophysically clarifying the structural basis of *h*AK1 catalyzing ADP into ATP and AMP, and, with the critical discussions above, it is necessary to address the atom-level revelation of the molecular mechanism describing the dynamics of *h*AK1 binding the substrate. To this end, molecular docking of *h*AK1 and its substrates as well as relevant molecular dynamics simulation have been included in our future plan and are actually under our investigation, which will facilitate further understanding of the molecular mechanism of *h*AK1 functions.

In summary, our study is beneficial to further understanding the role of conformational fluctuations involved in the enzymatic activities of *h*AK1, and provides insights into contributions of internal motions on different timescales to conformational changes induced by Mg^2+^-ADP binding.

## Figures and Tables

**Table 1:** NMR restraints and structural statistics for *h*AK1.

NMR Distance and Dihedral Constraints	Values
Total unambiguous distance restraints	2603
Intra-residual	884
Sequential (|i–j| = 1)	728
Short-range (2 ≤ |i–j| ≤ 3)	388
Medium-range (4 ≤ |i–j| ≤ 5)	144
Long-range (|i–j| > 5)	459
Total ambiguous distance restraints	402
Total dihedral angle restraints	350
φ (backbone dihedral angle C_i-1_–N_i_–C_i,α_–C_i_)	175
ψ (backbone dihedral angle N_i_–C_i,α_–C_i_–N_i+1_)	175
Structural statistics	
Mean restraint violations	
Distance restraint violations (>0.5 Å)	0.90
Dihedral restraint violations (>5°)	1.35
Average RMSD (Å) to mean structure (all residues)	
Backbone RMSD	0.57 ± 0.09
Heavy atoms RMSD	0.84 ± 0.06
Ramachandran plot statistics ^1^	
Residues in favored regions	89.2% ± 1.5%
Residues in allowed regions	10.6% ± 1.7%
Residues in generously allowed regions	0.2% ± 0.3%
Residues in disallowed regions	0% ± 0%

^1^ Analyzed with PROCHECK.

## Data Availability

The solution structure of *h*AK1 has been deposited in the Protein Data Bank with an accession code of 7X7S (https://www.rcsb.org/structure/unreleased/7X7S accessed on 10 June 2022).
